# The Vagueness of Integrating the Empirical and the Normative: Researchers’ Views on Doing Empirical Bioethics

**DOI:** 10.1007/s11673-023-10286-z

**Published:** 2023-11-08

**Authors:** T. Wangmo, V. Provoost, E. Mihailov

**Affiliations:** 1 Institute for Biomedical Ethics, University of Basel, Basel, Switzerland; 2 Bioethics Institute Ghent, Ghent University, Gent, Belgium; 3 Research Centre in Applied Ethics, Faculty of Philosophy, University of Bucharest, București, Romania

**Keywords:** Empirical bioethics, Integration methods, Reflective equilibrium, Qualitative study, Empirical research in bioethics, Interdisciplinary bioethics

## Abstract

The integration of normative analysis with empirical data often remains unclear despite the availability of many empirical bioethics methodologies. This paper sought bioethics scholars’ experiences and reflections of doing empirical bioethics research to feed these practical insights into the debate on methods. We interviewed twenty-six participants who revealed their process of integrating the normative and the empirical. From the analysis of the data, we first used the themes to identify the methodological content. That is, we show participants’ use of familiar methods explained as “back-and-forth” methods (reflective equilibrium), followed by dialogical methods where collaboration was seen as a better way of doing integration. Thereafter, we highlight methods that were deemed as inherent integration approaches, where the normative and the empirical were intertwined from the start of the research project. Second, we used the themes to express not only how we interpreted what was said but also how things were said. In this, we describe an air of uncertainty and overall vagueness that surrounded the above methods. We conclude that the indeterminacy of integration methods is a double-edged sword. It allows for flexibility but also risks obscuring a lack of understanding of the theoretical-methodological underpinnings of empirical bioethics research methods.

## Introduction

Empirical bioethics is an interdisciplinary activity that centres around the integration of empirical findings with normative (philosophical) analysis (Ives, Dunn, and Cribb 2017). Mertz and colleagues (2014) posited that “empirical research in EE [empirical ethics] is not an end in itself, but a required step towards a normative conclusion or statement with regard to empirical analysis, leading to a combination of empirical research with ethical analysis and argument” (p. 1). Thegrowth of this field is often attributed to a dissatisfaction with a purely philosophical approach, perceived as being insufficient to address bioethical issues (Hedgecoe 2004; Hoffmaster 2018) and hence a belief that an empirically informed bioethics is better suited to deal with the complexity of human practices. A consensus paper put forward by European empirical ethics scholars aimed to reach standards of practice for those working in and wanting to do empirical bioethics (Ives, et al. 2018). Concerning integration, the standards included the need to (1) clearly state how the theoretical position was chosen for integration, (2) explain and justify how the method of integration was carried out, and (3) be transparent in informing how the method of integration was executed.

Despite consensus that empirical research is relevant to bioethical argument (Mihailov, et al. 2022; Musschenga 2005; Sulmasy and Sugarman 2010; Rost and Mihailov 2021), integrating empirical research with normative analysis remains challenging. An often and long discussed way of integration is the (wide) reflective equilibrium (Daniels 1979), which has been tailored to serve empirical bioethics projects by several scholars (Ives and Draper 2009; Van Thiel and Van Delden 2010; de Vries and van Leeuwen 2010). Briefly, (wide) reflective equilibrium is a two-way dialogue between ethical principles/values/judgement and practice (empirical data). It is carried out by the researcher, “the thinker.” In this process, the thinker goes back and forth between the normative underpinnings and empirical facts (data available from the study or other sources) until he or she can produce moral coherence (an “equilibrium”).

A systematic review of integrative empirical bioethics identified thirty-two methodologies (Davies, et al. 2015). Amongst others, these include (wide) reflective equilibrium (Ives 2014; Van Thiel and Van Delden 2010; de Vries and van Leeuwen 2010), dialogical empirical ethics (Widdershoven, Abma, and Molewijk 2009; Abma, et al. 2010), reflexive balancing (Ives 2014), integrative empirical ethics (Molewijk, et al. 2003), hermeneutical approach to bioethics (Rehmann-Sutter, Porz, and Scully 2012), symbiotic ethics (Frith 2012), and grounded moral analysis (Dunn, et al. 2012). Davies and colleagues (2015) categorized the identified methodologies into, inter alia, (1) dialogical, where there is a reliance on a dialogue between the stakeholders (e.g., researchers and participants) to reach a shared understanding of the analysis and the conclusion (e.g., inter-ethics); (2) consultative, which comprises analysis of the data by the researcher, who is the external thinker and works independently to develop a normative conclusion (e.g., reflexive balancing, reflective equilibrium), and (3) those that combine the two (e.g., hermeneutics).

The wide variety of integration methodologies available illustrates considerable uncertainty about the particular aims, content, and domain of application (Davies, et al. 2015; Wangmo and Provoost 2017). Furthermore, the steps that guide the integration process are often unspecific (Davies, et al. 2015; Huxtable and Ives 2019). For example, if an ethicist acts as facilitator and applies ethical theory to enrich the dialogical process for decision-making in concrete situations (Abma, et al. 2010), one may wonder whether the application of ethical theories was up to the subjective appreciation of the ethicist. In reflective equilibrium, there are pressing issues of how much weight should be given to empirical data and ethical theory. The existing methodologies thus risk being frustratingly vague and insufficiently determinate in practical contexts (Arras 2009; Dunn, et al. 2008). All in all, the multiplicity of methodological paths and their lack of clarity gives rise to a debate about appropriate methodologies (Hedgecoe 2004; Ives and Draper 2009; Ives, Dunn, and Cribb 2017).

In a survey of bioethics scholars in twelve European countries, Wangmo and Provoost (2017), found that one-third of the respondents (total respondents N = 200) attempted to integrate the normative with the empirical. Their findings indicate that not everyone in the field of bioethics did or intended to engage in this kind of interdisciplinary work. A reason could be the methodological diversity and complications pointed to above. It is of importance to further clarify and, where necessary, develop (new) integration methodologies that address the needs in the field. In this explorative qualitative study, we set out to investigate how researchers perform the integration of empirical data with normative analysis and how they evaluate that process. Our hope is to learn from the experiences and reflections of researchers who engaged in empirical bioethics research and to feed these insights from practice into the debate on methods.

## Methods

### Sampling and Study Participants

To form our participant sample pool, we conducted a systematic search of peer-reviewed publications in two databases—PubMed and SCOPUS—and used the following key terms: “Empirical Bioethics” OR “Empirical Ethics” OR “Interdisciplinary Ethics” OR “Interdisciplinary Empirical Ethics” OR “empirical-normative” OR “normative-empirical” OR “Empirical research in Bioethics.” The literature search resulted in 334 results, from which we removed 143 results because they were duplicates or did not match our inclusion criteria. A sample pool of 191 papers were left. A separate Google Scholar search using the same terms lead to thirteen extra papers, resulting in a total sample pool of 204 papers.

Starting from this sample pool, we first aimed for a maximum variation sample of scholars according to the type of paper they had authored. Therefore, the 204 results were categorized into three groups: (a) Empirical: ninety-four; (b) Methodological: seventy-four; and (c) Empirical-Argumentative: thirty-six. Empirical papers were those that used purely empirical social science methodology. The methodological papers were those that discussed and/or used empirical bioethics research. Empirical-argumentative papers were those that produced empirical results along with an attempt to use them in an argumentative manner to make certain claims. These three categories were ordered alphabetically to allow simple random selection of the first authors of those included publications. Secondly, we also purposefully selected papers to aim for a balanced distribution of male versus female scholars. We carried out two rounds of selection which identified first authors of eighty-five publications who were invited to participate in our study. A total of twenty-four scholars agreed to participate. We interviewed two additional participants who were referred to us by a participant. See table 1 for participant information.

**Table 1:** Participant information

Total participants N = 26	
Gender	14 Female 12 Male
Experience	17 Senior researchers (assistant, associate and full professors, senior lecturers) 9 Junior researchers (PhD students and post-docs)
Expertise classification	8 Empirical ethicists (EE) 11 Empirical researchers in bioethics (ERB) 6 Social scientists working on ethical topics (SSE) 1 Theoretical ethicist (TE)
Region where the participants are located	7 North America 1 South America 14 Western Europe 2 Asia 2 Australia

### Data Collection

All selected first authors received an email from EM informing them about the study, its purpose, the researchers, and the voluntary nature of the study. All non-responders received one reminder. No incentive was given to participate in the study. The interviews were carried out using Zoom in light of the pandemic and because our participants were from different countries. The interviews were completed between April 2020 and January 2021 and were on average sixty minutes long (range forty-five to ninety minutes).

To structure the discussion, we used an interview guide composed of three sections. The first part of the interview was geared towards generally understanding the type of research carried out by the participants. Therefore, this part of the interview was not limited to the research presented in the paper via which they were selected. The second part aimed at their attitudes towards the purpose of empirical research in bioethics, using a series of eight statements to which they were invited to respond (Mihailov, et al. 2022). The third section sought participants’ experiences of doing empirical bioethics (i.e., integration), the advantages and challenges to carrying out empirical bioethics study, and their views on the empirical turn in bioethics. During the data collection process, the research team met twice to discuss the interview guide based on reading two of the first four interviews. This resulted in minor adjustments to the interview guide. For the interview guide and further information on the study method, please refer to the first paper from this project (Mihailov, et al. 2022).

### Data Analysis

Audio recordings were transcribed verbatim. All anonymized transcripts were imported into qualitative data analysis software, MAXQDA. Two authors (EM and TW) carefully read and coded several interviews independently and discussed the coding process and code labels used for the entire data corpus. This pre-coding followed a thematic analysis (TA) framework (Braun and Clark, 2006; Guest, et al. 2012) in light of its fit with the explorative nature of the overall project. Thereafter, a more specific analysis of the data related to integration methods took place in order to meet the aim of this paper.

The first author created and analysed a data set pertaining to participants’ experience, opinions, and their use of particular methods of integrating the normative and the empirical. Themes and sub-themes were developed based on authors’ discussion of the data related to the integration process. Using these themes and sub-themes, TW drafted the study results in a detailed and descriptive way for the co-authors (VP and EM) to gain the richness and depth of this specific content. After several rounds of iterations and discussions among the authors (process described in the next paragraph), we agreed on the result interpretations as presented in the next section.

Briefly, our analytical approach combines TA with a hermeneutics of faith or empathy and a hermeneutics of suspicion. Such approach has been used in other studies (Huxley et al. 2011). Whereas a hermeneutics of faith aims at better understanding what the participant described, a hermeneutics of suspicion aims to find out hidden or latent meanings. Our team integrated two types of hermeneutics that were reflected in the researcher roles: a hermeneutics of faith or empathy (EM, the interviewer and TW, the first author), a hermeneutics of suspicion (VP) and a mixture of both (TW). Integrating various analytic roles in one team has the advantage that different readings of the data can be used to challenge each other’s views, whilst still keeping track of those different interpretations. In the results section, these layers of interpretation are interwoven. We start with interpretations close to the participants’ accounts (the first two themes predominantly resulted from a hermeneutics of faith, where we also added critical notes at the end). As the results section progresses, critical interpretations that go beyond the data surface are given more weight (hermeneutics of suspicion). At the same time, we simultaneously keep underlining the scholars’ experiences in their own terms. We present data as block-quotes to support our analysis. Shorter expressions of the participants are given in the text using italic print between quotation marks.

### Ethics Approval

The study was approved by the Research Ethics Commission of the University of Bucharest. All participants provided their informed consent to participate in this study and to record their interviews.

## Results

We identified four themes related directly to our research question. The first theme “the back-and forth methods” relays the scholars’ accounts of using a reflective equilibrium method or similar. The second theme “collaboration as doing integration” deals with dialogical methods and the views of scholars who thought that collaboration was a better way of organizing integration. In reporting these two themes, we also illustrate the inherently vague manner in which the participants discussed their use of integration methods. Both theme labels were also chosen to reflect the simplified way several of the scholars conveyed their integration process. Thereafter, we continue with two additional themes, where we focus in on these accounts of participants’ chosen methods and how they were used. For this, we first present the theme “Integration as inherently ingrained from the start of the project; but is it integration?” In this theme, we start by critically looking at participants’ process of how the integration is done. Finally, we move further to unpack the ambiguity with which some participants spoke of engaging with these methods. In the theme “the integration method as a particular opaque intelligence” we highlight participants’ plea for creativity and flexibility. Here we note that although the participants are making a good point, this plea may at the same time reveal hesitance and uncertainty in talking about how they chose and applied the method they used.

### Theme 1: The “back-and-forth” methods

Several participants described their method as cyclical and included terms like “back-and-forth” between the conceptual framework and the empirical data. They alluded to their method as reflective equilibrium. Here, the participants noted that their research begins with a conceptual understanding of the ethical issues relevant for the topic or question. This was followed by the collection of empirical data based on the ethical concern teased out from conceptual work and going back to the conceptual to evaluate how it must be changed or adapted. Important in this backward and forward process was the notion of “*revising*” the theory and that this was an iterative process.

While doing the back-and-forth method of integration, one participant distinguished the normative and the empirical work, with the former being the core and the empirical elements being used to shape the normative concept. This reflective equilibrium method was also seen as a way of trying to understand why practice and theory are different; hence, it includes the need to go back-and-forth iteratively between what is happening in practice and why it does or does not conform to what is set out in theory.

My approach would be to start with the normative bit. Do(ing) research around that area. Have that firmly consolidated. With that, I could develop the empirical research bit: method, structure, instrument, population, whatever ... the design of the empirical bit. But probably that—the ongoing findings from this empirical bit, empirical research—would be continuously informing the normative bit that I already had then. And—as I mentioned before—for the output and the final outcomes, I think that probably starts by seeing how the empirical changed the shape of this normative “stone” [laughs]. (P18, SSE)

I think it’s kind of a reflective equilibrium thing going on and ... if it turns out that people who are on the front lines making certain kinds of moral decisions systematically think about a case a certain way, and that’s different, you know, they are sensitive to factors that maybe my theory thinks shouldn’t be important, it’s not obvious what should happen. Maybe I need to update my theory …. Or it might be that I come up with an account of why it is that they are systematically wrong, that their intuitions are corrupted in some way, or they’re responsive to factors that shouldn’t be normatively relevant. (P9, EE)

The iterative process was also seen as something that cannot be set into stone since one may have to go through several rounds of going backward and forward. Thus, a participant said that although this method is in essence a simple one, it cannot be recipe-like. This method was described as a creative process, explicitly set apart from empirical methods that follow a strict and preset schedule.

You know, this isn’t like science, where, you know, you have this type of data, do this statistical step, and follow x, y, z .... It is a creative process. You do your conceptual work; you look at the data. “No, that doesn’t work. Something’s not right. Doesn’t fit.” You go back to your concepts, reorganize them, look at your data again and other information you might have. So, it’s this iterative process of interpreting, reinterpreting data—you might have to go and seek more information, you know, if there’s certain gaps in what you ... to solve certain dilemmas you come up with. But yeah, I mean, it’s that simple. You just ... look at your data, try to ... gain meaning from that data and then conceptualize it and keep going backwards and forwards. (P7, ERB)

Within the participants’ accounts of doing such “back-and-forth” integration work, we were surprised by how often their descriptions expressed hesitance and uncertainty. This vagueness becomes clearest in this part of the discussions where an actual method of doing empirical bioethics was described. It was evident in the use of language such as *“it’s kind of a […] thing,”* and *“a bit of*.*”* Also, the participants used expressions such as *“trying”* and *“we reflect a bit and balance a bit”* when explaining how they used the method. These wording suggest a lack of confidence towards their own role in the methodological process.

I think basically my advice is some kind of evaluation of judgement is a normative one, philosophical normative one, but I try to use empirical [data] as in some kind of understanding, or I try to apply those normative into the practice, and also when the real or the empirical data, empirical knowledge, has some different implication or different meaning, then I could go back for my normative one. So, it’s kind of the reflective equilibrium thing. (P25, ERB)

So, what we normally say is that we use a bit of the method of reflective equilibrium, trying to combine all kinds of considerations [of the people you are studying or the issue of the study], and norms and values and principles and professional norms and individual norms. And try to mix those and weigh those and come to an equilibrium. (P19, ERB)

### Theme 2: Collaboration as doing integration without a distinct integration method

Some participants said that integration can be done through collaboration, in which two or more researchers with different skills (normative analysis and empirical method) would come together to formulate the research question and conduct the study. Participants reporting this mode of integration used a dialogical encounter. It was advised that the researchers with different backgrounds should know each other’s trade and work closely together, although in some ways also staying distinct. Calling for collaboration, one participant felt that although each researcher within their respective disciplines needs their own methods, there is no particular need for a standardized overarching method.

Well, first of all it’s an interdisciplinary work. So, you need the methodology, and you need the experts in their fields. For the empirical part you really need experienced social scientists, who know how to do empirical research in a valid manner. And for the normative part you need philosophers and people who are used ... are familiar with how to approach a normative question. And I think what is also important is that they know from each other and their different methodology and work. … So, integration sounds a little bit as if all things come together ... kind of a ménage. But there ... I see it more as staying distinct but working very closely and interactively together. But still with different methodology and [remaining] aware that they are different. (P20, ERB)

One participant explained this collaborative integration as a communicative process where the normative conclusions drawn are the result of discussions with the study participants, stakeholders, and even journal readers and other audiences. This participant’s collaboration method made a clear differentiation between the empirical and the theoretical parts. That is, the empirical phase stops after finishing the data collection and the (first-level) interpretation of those collected data. Thereafter, the empirical results are taken through a process of discussions with different stakeholders, a collaborative process that in theory is unending as it continues even after the publication of the study findings.

So, we were very interested in how they [their participants] narrate what they experience, and we saw, that [they] have typical […] narratives, with which they identify. […] That was the empirical approach and then at the end there was another [approach] between the results from the empirical part and the more theoretical or bioethical discussion, where we had regular interactions with the two parts of our team and some of the members, myself included, per parts of the empirical theme and of the theoretical theme and so we had this exchange of perspective and that led then to the publications. It’s a communication process. I think bioethics is always a conversation, also when we just write up papers, we are in a conversation, just one step in a conversation. So, your question how to integrate, is how to proceed in more comprehensive conversation with the audience, the readers of our papers, we are addressing. (P4, EE)

What these participants relayed is that the integration occurred through the process of collaboration. In these accounts, there is no specific integration method used during this collaboration and no plea for an overarching method of integration. Another way of stakeholder collaboration leading to integration was described as dialogical encounter during workshops. Here the key idea was that the research team along with their invited experts deliberate on the aggregate findings and reach a consensus as to what could be the key message of the overall work. Here again, we notice how no specific method (used during such collaboration) was brought forward.

Yeah. We tend to do a little bit of reflection ourselves on the data to come up with a conceptual map or model or policy recommendations and then we try to iterate that with the group, because we realize that, you know, we have a responsibility together. Right? And so balancing our ideas offered people ... it’s a good way of assessing whether ... when we are making the shift from what the “is” is to perhaps what the “ought” should be. Having different perspectives there is important. And we do that and depending on the project, sometimes we built in a formal consensus process, another time we just want to test our ideas to see how they ... If other people endorse them or can make some suggestions to improve them. (P3, ERB)

I think ... I don’t think we need one [a specific method]. I really, I don’t. I don’t actually think we need one. Because a lot of people do a lot of good work—either empirically or normatively—and there are people who get along and so ... I think that is the empiricist and the normative […] and I also very hate to “pick” ... I think we have a lot of people who do both really well. But what I WISH ... is that instead of looking for a recipe to be able to integrate ... that people with different expertise would just work together more often. (P15, ERB)

Overall, we saw a similar vagueness in their description of the “how” of integration. For example, in the quote above, the participant talks of *“balancing”* that is done among the invited stakeholders as part of their discussion. It remains unclear how exactly such collaboration occurs and how to confirm the value of the outcomes reached. Also in this quote, we note the language of indeterminacy we described above (e.g., *“try to iterate”*).

### Theme 3: Integration as inherently ingrained from the start of the project; but is it integration?

Several scholars did not consider it necessary to use a specific method of integration. They reported that, for them, the normative and empirical parts of a study are interwoven within the different phases of the research process. According to these participants, the normative and empirical cannot be teased out. This is because these are inherently linked from the start of the study, with the research question and the research project being, in and of itself, normatively oriented. The empirical and the normative are constantly informing one another: “*you cannot separate the normative from the empirical. When doing empirical work, you already do a lot of normative work as well. So yeah it’s for me it’s integrated* anyway” (P12, EE). Adding to the above quote, the same participant stated, “*No, it’s always both [normative and empirical], you cannot separate actually. But it also depends on what you understand as normative analysis of course*.”

However, some scholars who felt that they were also doing this type of integration in empirical bioethics, to our view, are mistaken. This is because they were either (1) describing what looked to be purely theoretical research activities or (2) presenting what looked to be purely empirical activities as both empirical and normative. For instance, one participant argued that the normative and the empirical are not distinguishable in that there is no separation between the normative and empirical. This scholar talked about a feature of this approach, where “*no data is gathered*” as it was a process of doing philosophical work in context. The claim was that the entire research is situated in the world of “oughts,” thereby making it possible to come to an “ought” statement without having to trouble oneself with the is-ought gap. What this scholar sees as “integration” looks like context-sensitive normative argumentation.

So, the integration account is basically the production of a certain kind of an argument in a certain kind of context. And that’s why the integration that I defend, I guess, is, it’s so, it’s about normative reasoning of a certain kind, taking place in a certain kind of context, in situ. Which is why I resist the idea of, as seeing descriptive and normative phases. If you take that view, you’re basically saying something I think more profoundly about how, that data can produce an understanding of the ethics or something like that or that data can profoundly impact on our political positions. I don’t think that’s what the data is doing, insofar as what data is doing on my account on integration, it’s much more about how we can make better, how we can make arguments that have a particular kind of fall. (P6, EE)

A few other participants’ empirical bioethics work seemed to us as merely descriptive-oriented research activities on ethically relevant topics. One participant stated how the normative and empirical are not distinguishable and that somehow the analysis process is when normative thinking takes place. In this, however, no normative undertaking of the data was evident. Within their descriptions, we also found statements that conveyed vagueness in how this process of integrating the empirical and the normative was done. For instance, a participant regarded several parts of the research process, interpreting and discussing the research data, as normative in nature because it could not be disentangled from normative presuppositions.

Yeah. So, the way I do data analysis is by listening to the audio of interviews and also reading transcripts. And so ... often by the time I’ve gotten to the point of analysis I already have ... interpretative themes … So, it really is an integrated theoretical and empirical process. (P16, SSE).

I wouldn’t know how to distinguish the empirical and the normative because ... what you can do empirically is deeply dependent on ... normative ... presuppositions. Ehm ... and then of course, what you actually do when you ask people for responses, and when you do ... your statistical analysis, I mean that’s not […] that’s only partly normative in the epistemic sense, but not in the moral sense. Ehm so, that’s obviously empirical then. But again—as soon as you start interpreting and discussing the empirical results—you’re back in the normative arena so, that really goes hand in hand. (P23, TE)

In another example, participants explained how in a descriptive type of study on an ethical topic, the normative work still played a role by referring to a thematic map that was based on normative concepts. However, one could claim that by describing the normative part as doing “*an empirical analysis in an ethically relevant way”* they actually place this research activity fully within the empirical domain.

I mean, the normative and the empirical, what I actually, I’m not so much concerned with that question, even though that may be a little bit, um, bit weird. Um, I often think a little bit different, I think like what can I contribute for the empirical and what can I contribute from the applied ethics, perspective so to say. It doesn’t necessarily have to be normative, um, it just needs to be in the realm of ethics so to say, so again if I talk about [ethical topic of the participant’s research], I, I’m also just interested in what do they [researchers] think is their [values on the ethical topic], how do they frame their [value on the ethical topic], and by asking them about [the ethical topic] I ask them about their actions, what they do, why they do it, what is their normative basis, all those things, and by that I already ensure the ethical debate, to some degree. *(P5, ERB)*

If you are doing the interviews, I would say, this is more the point where you are on the empirical parts …. Though I would still say, it’s very helpful to have the normative background assisting, when you are doing the interviews and hearing out what are the normative interesting things that people say. So, still it is not completely gone, the normative background. When you are analysing the data, then I would say, you have the empirical part for one, because you have to do this in an empirically solid manner, but you also have the normative part included, because you want to analyse the data not just in a sociological way, but you want to analyse this in an ethically relevant way. (P2, EE)

### Theme 4: The integration method as a particular opaque intelligence

Within this theme, we illustrate how the vagueness in the methods used was more explicitly brought forward as a feature of these methods. Participants who have done empirical bioethics or sought to do it described how one can go from one step to the next to reach the normative conclusion. Their use of terminologies to describe this vague process pointed to something mysterious: an “*opaque A.I.*” and a “*big leap*.” The process was seen as something that was difficult to explain. One participant claimed it could not be put into precise methodological rules. We pointed to this argument above when we reported the case participants made against recipe-like methods. Here, the participant explicitly raised the view that this process remains open to post-hoc justification.

What does integration really mean? How do you articulate this—kind of—magic box, where data goes in and then you come out conclusions?. It’s a particular opaque A.I., where you—kind of—plug in the data and this conclusion comes out. … And, that’s not a transparent process, we don’t know how our brains work, we don’t know how we make connections. So all we can do is perhaps be transparent about the steps we’re taking to get the information, be reflexive about how we use information, and then articulate the reasons for our conclusions. But I think—as I said earlier—there will always tend to be post-hoc justifications. (P22, EE)

And then the big leap ... and the big leap is probably the one that you are curious about. The big leap toward what is the good thing to do. … But yet again, I have always thought that that methods [reflective equilibrium] falls short in giving clear sight of the black box, of the end, of the conclusion, ... I don’t have an answer whether or not we really get a clear view what happens when we take the “jump” from what we see, what we think, towards what we think would be the right thing to do, what we ought to do. (P19, ERB).

Accounts where we saw this vagueness presented as a feature of the method also expressed a need for a creative process that would require some flexibility. In the same line another participant noted: “*I feel that if we did have a recipe for integration, it would almost be sad ... people might feel that they are finding the ‘holy grail,’ but then you limiting yourself to just one way of thinking”* (P15, ERB). Several participants underscored the need for flexibility and not to be restricted by too many rules. They said that much of empirical bioethics seeks to integrate work from two disciplines that have indeterminate processes, i.e., qualitative research and theoretical ethics. They thus emphasized the challenges of articulating two methods that are themselves opaque into one that is not.

And I think qualitative researchers have been ... struggling with this for a long time, and I think a lot of what we’re doing now mirrors the difficulties that qualitative researchers have been having—particularly in medicine—where they’re being challenged to explain a method. … And we have to explain method, but you can’t explain how your brain got there. With empirical bioethics, we’re working with qualitative research AND we’re working with ... theoretical ethics, so it’s doubly challenging to articulate two uhm very opaque processes. (P22, EE)

## Discussion

In 2015, Davies and colleagues summarized thirty-two empirical bioethics integrative methodologies that combine normative analysis and empirical data obtained using social-science research. Following this, scholars have discussed the integration of the normative and life sciences research (Mertz and Schildmann 2018), using critical realism in empirical bioethics (McKeown 2017), and integrating experimental philosophical bioethics and normative ethics (Earp, et al. 2020; Mihailov, et al. 2021). In line with the systematic review of empirical bioethics methodologies’ two broad categories of dialogical and consultative processes of integration (Davies, et al. 2015), our participants indicated two familiar approaches. The first one is based on a reflective equilibrium–type process, and the other, an interdisciplinary collaboration between and among different stakeholders.

In addition, several participants suggested integration was inherent with the normative and empirical intertwined within the overall research process. Our participants’ accounts of inherent integration shared some similarities with, for example, moral case analysis (Dunn, et al. 2012), integrated empirical ethics (Molewijk, et al. 2003), and dialogical empirical ethics (Landeweer, et al. 2017; Widdershoven, et al. 2009). The shared similarities were in the sense that there were no separate normative and empirical parts to be distinguished in a project and that the project itself was normatively oriented. However, we should be critical of this view. The mere fact the empirical and the normative is inseparably intertwined throughout a research process does not mean (1) that these claims cannot be conceptually separated and (2) that such a method is free of methodological concerns. For instance, there would still be the need to specify what moral principles demand in a particular situation, decide which ethical theory to use, or make normative judgements with the help of empirical data (Frith 2012; Salloch, et al. 2015). Apart from that, several of these “inherently integrated” methods lacked a clear normative side and the enterprises described seemed purely empirical. Upon closer analysis, one could interpret some of the accounts of “integration was always inherently present” as a way of avoiding looking into the black box.

Furthermore, within these “inherently integrated” approaches, a few scholars described their descriptive research on ethical issues as empirical bioethics. Based on the available definition of empirical bioethics (Ives, Dunn, and Cribb 2017; Mertz, et al. 2014) and the standards offered by Ives and colleagues (2018), the works of these participants would thus not count as empirical bioethics. This is because there was no evidence of any integration happening. In our opinion, this mismatch between the practice of some scholars and what is “agreed” to in the literature as empirical bioethics may be pointing to the fact that empirical work in bioethics is in essence heterogeneous (Ives, Dunn, and Cribb 2017; Mertz, et al. 2014). For one, it is possible that scholars look at their projects as fitting an empirical bioethics because they start from research questions relating to the normative and because their projects, even with purely descriptive parts (and papers), are aimed to eventually lead to normative conclusions. But also in that case, we need to be clear about the nature of such particular (sub)projects and about the absence of integration efforts in these parts. Second, it is possible that scholars have different perspectives on the matter than the one expressed in the standards paper (Ives, et al. 2018). In that case as well, these must be brought out in the open. Third, some scholars may simply be mistaken when they consider their projects to be empirical bioethics. Their mistaken belief might be based on the idea that the empirical findings were at some point integrated in normative reasoning, which results in a normative claim. This simply might not be the case. This then, more than anything, would point to the need for transparency about and agreement on the use of methods. A heterogeneity of approaches in the field should be applauded. However, for all of them, we need to be able to identify where and how the integration happens. In the remaining part of this discussion, we focus on the overall vague manner in which our participants talked about their methods and what that implies for the field of empirical bioethics.

### Vagueness of Integration Methods Used

Reflective equilibrium, broadly construed, is a deliberative process that seeks coherence between attitudes, beliefs, and competing ethical principles (Daniels 2020). A standard objection against reflective equilibrium methodology is that it is insufficiently determinate in practical contexts to be action-guiding or to help decide between conflicting views (Arras 2009; Paulo 2020; Raz 1982). The iterative process of going back-and-forth between the normative and the empirical to come to a coherent account, similarly, is fraught with indeterminate indications. The way study participants relayed their approaches and explained their practices underscored the vagueness they felt. It further showed the difficulties even scholars with expertise in using these methods had in illustrating the “how” in an exact manner.

Such vagueness was also evident in collaboration methods of integration reported by our study participants. This collaboration involves an iterative and deliberative process of sharing information and engaging with different perspectives (Rehmann-Sutter, et al. 2012). It requires ongoing dialogue between social scientists and bioethicists. Their practical know-how guides the conclusion about the normative significance of empirical data. Even though the experience and implicit know-how of the experts can be rich in content and varied, how the communication process is done and who decides the outcome often remains indeterminate. This was noted in the voices of our participants.

The difficulty in clearly explaining the “how” of the integration process is something that researchers who have carried out an integration or wished to do so are likely to be familiar with. Several scholars have pointed to this unclear process as well (Ives and Draper 2009; Mertz and Schildmann 2018; Strong, et al. 2010). One explanation for this finding may be that, given the numerous tailored versions of the reflective equilibrium methodology for empirical bioethics (de Vries and van Leeuwen 2010; Ives 2014, Ives and Draper 2009; Van Thiel and Van Delden 2010; Savulescu, et al. 2021), there may be confusion surrounding how to make a choice and how to implement it in practice. As noted earlier, there are many available empirical bioethics methodologies (Davies, et al. 2015), and it has been suggested that each researcher could be using his or her own version (Wangmo and Provoost 2017). This situation, to us, points in two directions. First, it may convey a general need to remain flexible and open to creativeness, key components of the normative reasoning that is central to the integration method. We may thus have to stop looking for a method that is akin to empirical standards, especially those of quantitative methods, and recognize that the empirical and normative integration is in many ways a normative enterprise, which does not follow an exact method. Second, the wide variation of approaches makes it even clearer that we need to seek more methodological clarity on the overarching level. This is where the debate on standards (Ives, et al. 2018), for instance, has been an added value. It allows for heterogeneity while at the same time striving to create more clarity. In fact, we point out that the integration methods are inherently indeterminate and that this is a good thing. That said, an acceptance of the indeterminate character of this integration does not absolve us from the need to identify the foundations of what we are doing in a theoretical-methodological way.

The study findings confirm the image of an indeterminate process. As research on this topic is developing, it is ever more clear that the scholars involved come from a wide variation of disciplines. This is another argument as to why this indeterminate character is indispensable. The findings thus substantiate what has already been written about the indeterminate status of the methods used in empirical bioethics (Arras 2009; Davies, et al. 201^5^; Dunn, et al. 2008; Huxtable and Ives 2019), despite efforts to delimit and standardize empirical bioethics work (Mertz, et al. 2014; Ives, et al. 2018). One way of reading the vagueness we encountered is the scholars’ struggle to explain their own integration process, and perhaps even a lack of full comprehension of that process. Another interpretation is one that is in line with the wish for creativity and flexibility, and a level of indeterminacy in the methods we look for, namely an expression of leaving things open. Creativity can be a medicine against the belief that precise and transparent standards can account for such a “maze of interactions” (Feyerabend 2010) between experts with fertile know-hows. Too much standardization misses how particular research situations inspire novel ways of seeing the ethical relevance of empirical data. We should nevertheless be aware that the indeterminate nature of any integrative methodology makes it subject to risks of post-hoc rationalizations and motivated reasoning (Ives and Dunn 2010; Mihailov 2016). In the end, demands for creativity—however valid—should go hand in hand with demands for a thorough theoretical foundation as well as practical understanding of the method at hand.

### The Normative Nature of Integrative Methodologies

Reflective equilibrium is a deliberation method that helps us come to a conclusion about what we *ought* to do (Daniels 1996; Rawls 1951, 1971). If we describe the integration process only in terms of going back-and-forth between data and theory, or in terms of collaboration between different experts, we risk obscuring the normative nature of using empirical data to help elaborate ethical prescriptions, which is the goal of doing such an integration (Ives and Draper 2009; Mertz, et al. 2014). Researchers often talk about integration as if it is a process half empirical and half normative or something that just needs normative reasoning alongside empirical data. But the very act of integration is normative in nature. While facts are essential for addressing bioethical issues, the task of integration ultimately depends on normative assumptions about the normative weight of moral intuitions.

Our data show that many of our participants rely on a reflective equilibrium characterized in their explanations mostly by *moving back-and-forth* between empirical results about moral attitudes and intuitions. Although the cyclical thinking is an important part of reflective equilibrium, there is more to it. Often, however, our participants did not move beyond this aspect. Ideas of coherence between moral intuitions and moral principles, and the fundamental willingness to adjust moral principles in light of what we discover were rarely touched upon. Perhaps what we see here is that several study participants embarked on an intuitive account of a—sometimes simplified—reflective equilibrium inspired methodology. At least in the interviews, it was not shown that they were fully aware of theoretical commitments to coherence, giving normative weight to moral intuitions, and screening them for bias.

The need to clarify the essential normative nature of integration appeals to normatively trained bioethicists, who may be in a better position to debate and assess how empirical input should be integrated into normative recommendations. We are not claiming that bioethics should be the arena of philosophers. Empirical research in bioethics is widespread (Borry, et al. 2006; Wangmo, et al. 2018), and scholarly perceptions about who belongs in the field are no longer exclusivist. There is thus a need to look at empirical bioethics projects in a broader way, including studies where empirical data are gathered but not used directly as part of a normative argumentation. Such empirical data may thus contribute to a larger body of work aimed at reaching normative conclusions. They can include, for example, empirical studies that explore stakeholders’ views relating to bioethical matters and explain how people arrive at certain reasoning patterns or studies that reveal the lived experience of stakeholders and explore how moral questions are experienced in practice (Mihailov, et al. 2022). To our view, despite the central role of normative know-how to integration, this does not mean that integration efforts need to be exclusively the work of ethicists or that empirical researchers will be unable to engage in it.

### Limitations

Our findings are, first and foremost, not generalizable, as they are based on an exploratory qualitative study design. The data come from a small non-representative sample of researchers. Other scholars, with different or greater experience in using particular (interdisciplinary) integration methods may have different opinions. They could perhaps have provided us with more concrete information about the way they carried out such integration. Also, only one of our participants described him/herself as a normative researcher. It would have been interesting to have more participants who were normatively oriented to include their views on how empirical data can be of use to the adaptation or formation of normative recommendations. Second, we asked scholars to tell us the process they use in integrating the normative and the empirical. This is a challenge task in and of itself. Not only did the scholars have limited time for the interview, but also it is generally difficult to explain how exactly this process pans out post-hoc. We thus acknowledge that we presented the participants with questions which were in no way easy for them to address in a single conversation. Because we wanted to focus on the scholars’ own reports, we did not confront them with approaches adopted by others in as systematic way. We did not also engage in a critical assessment of the reported method at the time of the interview. It would be interesting for further research to include such an approach and, for instance, study this using focus group methods. Using confrontation with other approaches or other views could offer the opportunity for a more critical reflection. For this paper, however, we opted to enrich the ongoing debate first and foremost with the accounts of the scholars. Third, we underline that a minority of our participants had already published methodological papers related to empirical bioethics as evident from the EBE sample. We did not ask the scholars to discuss the method that they have written about or most liked, nor did we ask them to discuss the paper that led to their identification for this study. During the interviews, however, we sought to address acquiescence and social desirability by using Socratic questioning and probing, to provide time for participants to explain their method of integration.

## Conclusion: Ambiguity Waiting to Be Disentangled

We set out to find more about the “how” of the integration methods used by scholars in empirical bioethics. Our hope was to provide input for the ongoing debate on methods and perhaps even some practical support for those considering empirical bioethics projects. Although we shed some light onto the way integration methods were used by different bioethics scholars, we especially bring forth the vagueness and uncertainties in their accounts. The main challenge was not the heterogeneity of methods but rather the indeterminate nature of integration methodologies. On a practical level, this finding may express the need for flexibility and variation in approaches rather than a need for recipe-like instructions. Such a clear-cut method will likely neither be possible nor appreciated. Philosopher of science Paul Feyerabend once said that methodological rules “are ambiguous in the way certain drawings are ambiguous” (2001, 39). The ambiguity of integration methods does not make them less appealing, just as the ambiguity of drawings does not make them less beautiful. Therefore, we may be wiser to accept some degree of indeterminacy, while simultaneously striving for clarity and transparency in terms of the theoretical-methodological underpinnings.

## Data Availability

Anonymized data relevant to evaluate the results presented in this paper can be made available upon request.
